# The risk of attention deficit hyperactivity disorder symptoms in the adolescent offspring of mothers with anxiety and depressive symptoms. Findings from the raine study

**DOI:** 10.1192/j.eurpsy.2021.261

**Published:** 2021-08-13

**Authors:** G. Ayano, K. Betts, R. Tait, B. Dachew, A. Lin, R. Alati

**Affiliations:** 1 School Of Public Health, Curtin University, Perth, Australia; 2 National Drug Research Institute, Faculty Of Health Sciences, Curtin University, Perth, Australia; 3 Telethon Kids Institute, Universityu of Wetsren Austrlia, Perth, Australia

**Keywords:** Attention deficit hyperactivity disorder, anxiety, depression, offspring

## Abstract

**Introduction:**

While there exist some studies that explored the association between maternal anxiety and depressive symptoms and the risk of attention-deficit/hyperactivity disorder (ADHD) in early and late childhood, studies exploring the risk in late adolescence are however lacking.

**Objectives:**

This is the first study that aimed to investigate the association between maternal anxiety, depressive, as well as comorbid anxiety and depressive symptoms, and the risk of ADHD symptoms in late adolescence.

**Methods:**

We used data from the Raine Study, a birth cohort in Western Australia. The Depression, Anxiety, and Stress Scale (DASS) was used to assess maternal depressive and anxiety symptoms when the child was aged 10. Whereas, the DSM-oriented scales of the child behavior checklist (CBCL) was used to assess ADHD symptoms offspring in adolescents aged 17. Log-binomial regression model was used to explore the associations.

**Results:**

After adjusting for relevant covariates, we found an increased risk of ADHD symptoms in the adolescent children of mothers with anxiety [RR 2.84 (95%CI 1.18-6.83)] as well as comorbid anxiety and depressive symptoms [RR 5.60 (95%CI 3.02-10.37)]. No association was seen with maternal depressive symptoms.

**Conclusions:**

This study suggested that adolescent offspring of mothers with anxiety as well as comorbid anxiety and depressive symptoms had an increased risk of ADHD symptoms. Early detection and management for ADHD symptoms in children of mothers with anxiety and comorbid anxiety and depressive symptoms are needed.

**Disclosure:**

No significant relationships.

